# Drug Delivery Applications of Three-Dimensional Printed (3DP) Mesoporous Scaffolds

**DOI:** 10.3390/pharmaceutics12090851

**Published:** 2020-09-08

**Authors:** Tania Limongi, Francesca Susa, Marco Allione, Enzo di Fabrizio

**Affiliations:** 1 Dipartimento di Scienza Applicata e Tecnologia, Politecnico di Torino, Corso Duca Degli Abruzzi 24, 10129 Torino, Italy; francesca.susa@polito.it (F.S.); enzo.difabrizio@polito.it (E.d.F.); 2 SMILEs Lab, PSE Division, King Abdullah University of Science and Technology, Thuwal 23955-6900, Saudi Arabia; marco.allione@kaust.edu.sa

**Keywords:** drug delivery, three-dimensional porous scaffolds, electron beam melting, selective laser sintering, stereolithography, electrospinning, two-photon polymerization, osteogenesis, antibiotics, anti-inflammatory

## Abstract

Mesoporous materials are structures characterized by a well-ordered large pore system with uniform porous dimensions ranging between 2 and 50 nm. Typical samples are zeolite, carbon molecular sieves, porous metal oxides, organic and inorganic porous hybrid and pillared materials, silica clathrate and clathrate hydrates compounds. Improvement in biochemistry and materials science led to the design and implementation of different types of porous materials ranging from rigid to soft two-dimensional (2D) and three-dimensional (3D) skeletons. The present review focuses on the use of three-dimensional printed (3DP) mesoporous scaffolds suitable for a wide range of drug delivery applications, due to their intrinsic high surface area and high pore volume. In the first part, the importance of the porosity of materials employed for drug delivery application was discussed focusing on mesoporous materials. At the end of the introduction, hard and soft templating synthesis for the realization of ordered 2D/3D mesostructured porous materials were described. In the second part, 3DP fabrication techniques, including fused deposition modelling, material jetting as inkjet printing, electron beam melting, selective laser sintering, stereolithography and digital light processing, electrospinning, and two-photon polymerization were described. In the last section, through recent bibliographic research, a wide number of 3D printed mesoporous materials, for in vitro and in vivo drug delivery applications, most of which relate to bone cells and tissues, were presented and summarized in a table in which all the technical and bibliographical details were reported. This review highlights, to a very cross-sectional audience, how the interdisciplinarity of certain branches of knowledge, as those of materials science and nano-microfabrication are, represent a growing valuable aid in the advanced forum for the science and technology of pharmaceutics and biopharmaceutics.

## 1. Introduction

Recently, one of the main thrusts of the micro and nano technologies application in the biomedical and clinical field has certainly been observed in the pharmaceutical drug delivery technologies optimization. Whether it is based on active or passive drug delivery, the way in which drugs are delivered substantially impact their efficacy and toxicity affecting their biocompatibility, pharmacokinetics, and pharmacodynamics. Drugs and active molecules can be introduced into the body via a number of administration routes such as buccal/sublingual, nasal, ocular, oral, pulmonary, anal/vaginal, transdermal and parenteral drug delivery [1,2,3]. Since a high percentage of the active pharmaceutical ingredients settled by the pharmaceutical production are precluded for a classical administration route, due to their low bioavailability [4], novel technologies assist modern drug delivery. As a result, an increase is observed in the effectiveness and reduction of side effects of the formulations in relation to patient compliance and costs reduction. In the past years, many drug delivery systems as organic and inorganic micro- and nanoparticulated systems as nanoparticles, micelles, liposomes, extracellular vesicles, nanotubes, metal–organic frameworks (MOF) and hydrogels have been used to deliver drugs at their therapeutic concentration to specific cell types and tissues [5,6,7,8]. Both material and design should be taken into account when optimizing a drug delivery carrier able to guarantee tuneable release (sustained, controlled, or pulsed), to act as a temporary reservoir, to increase the solubility of hydrophobic formulations, to float in the gastrointestinal tract and to protect the biological cargo from degradation [9].

Porous carriers have been successfully used as drug delivery matrices for their surface properties, high surface area and tuneable pore dimensions [10,11]. According to their pore sizes, porous materials are classified into three different categories, namely microporous, mesoporous, and macroporous [9,12]. Microporous materials such as MOFs and zeolites, are characterized by a well-interconnected network of pores less than 2 nm in size and high thermal stability and catalytic activity [13]. In macroporous materials, pores dimension ranges between 50 and 1000 nm [14] while in mesoporous materials pore size is between 2 to 50 nm [15]. In more details, mesoporous materials with a narrow pore dimension distribution and high surface area can be considered valuable candidates in drug delivery applications [16,17]. In the wide category of mesoporous, many materials are included such as mesoporous silica, hydroxyapatite and carbon, hydrogel and nanogel, metal and metal-doped nanoparticles. These materials have great versatility since their actions can be regulated by tuning the chemical environment optimizing the loading and consequent release of the chosen drug [18,19,20]. The drug incorporation into a mesoporous material is usually carried out by embedding the matrix in a concentrated solution of the drug and by a successive drying step. The size of the absorbable molecule (from small active molecules to proteins) is related to the dimension of the pore, and generally, a pore/drug size ratio >1 allows the adsorption of active molecules inside the pores. By using polymeric structure-directing agents, varying the chain length of surfactant or solubilizing supplementary substances into micelles, mesopores sizes can be adjusted from some nanometres to several tens of nanometers [21].

Recent advancement in micro/nano-fabrication techniques, materials science, chemistry and pharmacology has allowed the development of a number of mesoporous materials for drug delivery application characterized by evident structural advancement such as tuneable pore sizes, different grade of skeleton rigidity and two/three dimensional (2D–3D) architectures arrangement [22,23,24,25,26].

Hard (nanocasting) or soft templating approaches are applied to produce ordered mesostructured porous materials. The templated synthesis usually requires three successive steps: template preparation, template-directed synthesis and template removal. Hard templating leads to very robust structures containing several constituents as carbon, and metals (oxides, nitrides and sulphides) [27,28]. It is a synthetic method based on the deposition of the targeted materials into the narrowed spaces of the template, resulting in a reversed copy of the mold. The pores of these templates are soaked with a precursor of the looked-for product (e.g., a metal salt for metal oxides) which is in situ thermally transformed to the final product. When the template is removed, mesoporous material remains as the negative replica of the hard template [29].

Soft-templating techniques allow direct synthesis of porous materials through block copolymers including blocks of ionic and non-ionic oligomers, amphiphilic surfactants employed as structure-directing agents (SDAs) and through the addition of precursors as metal salts for metal oxide nanomaterials and organosilanes or triethoxysilane for SiO_2_-based nanomaterials. Soft-templating techniques are those in which small sub-units self-assemble to define the final structure, which is an aggregate of these starting units, which are not embedded in other matrices or removed as in the techniques described above. Upon self-assembly in a solvent, a micellar structure is realized by the fact that the hydrophobic sides of the molecules of the amphiphilic surfactants point inward and the hydrophilic ones outward in case the solvent is polar, while the opposite occurs if the solvent is non-polar. After this step, micelles are functionalized on their external corona structure using functional groups, frequently polymeric oligomers. Finally, it is the cross-linking of these external terminations which assemble the micelles in a mesoporous superstructure [30].

Producing porous hierarchical materials by integrating macropores in mesoporous tools manifestly increases their practical drug delivery applicability since macropores increase mass transport decreasing diffusion restraints characterizing purely mesoporous materials, while the mesopores empower great surface area [31,32].

**Figure 1:**
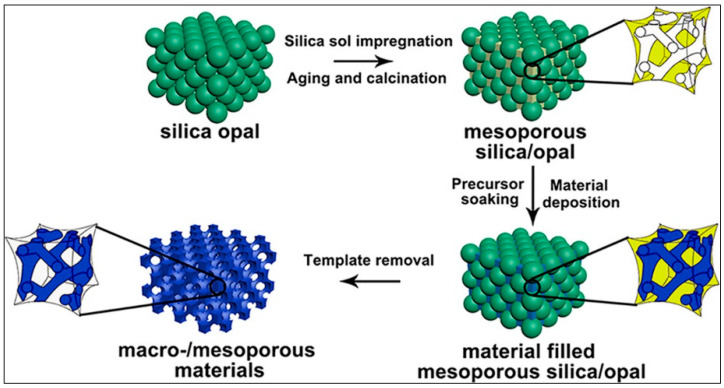
Schematic representation of 3D macro/mesoporous materials preparation reproduced with permission from [34], Chemistry of Materials, 2018.

Many methodologies have been optimized to engineer the hierarchically structured mesoporous solutions. The dual-templating synthesis method, applying colloidal crystal (opal) hard-templating and soft-templating techniques, is employed for realizing, as schematized in Figure 1, 3D macro/mesoporous materials for a wide range of applications, including the drug delivery ones [33,34].

## 2. 3D Printed (3DP) Mesoporous Scaffolds Fabrication Technique

The idea of realizing a macroscopic object via a bottom-up approach has been attractive for a long time but recently, the advancement of both the materials to be used and the techniques to be exploited have made possible the fabrication of 3D printers able to produce any shape in many different natural [35], synthetic, plastic and metallic materials, at variable size scales and with potentially very high accuracy in positioning [36,37,38]. This has pushed some researchers towards the idea to explore the possibility to use these techniques to realize solutions with different designs, characterized by being made of different types of mesoporous materials [21]. 3D porous substrates, used with or without further functionalization or engineering, are used more and more frequently in in vitro and in vivo drug delivery studies to assist cell growth or tissue regeneration ensuring the right degree of asepticity and differentiation [39,40,41].

2D and 3D printing tools are appealing for drug delivery applications since state-of-the-art equipment allows the deposition of liquid, gel, and solid constituents enclosing a wide range of pharmaceutics according to predefined schemes. The layer-by-layer assembling mode to print scaffold allows exact control of the design and of the geometry of the internal pores system, which consequently leads to tune the strength of the final products [42,43].

**Figure 2:**
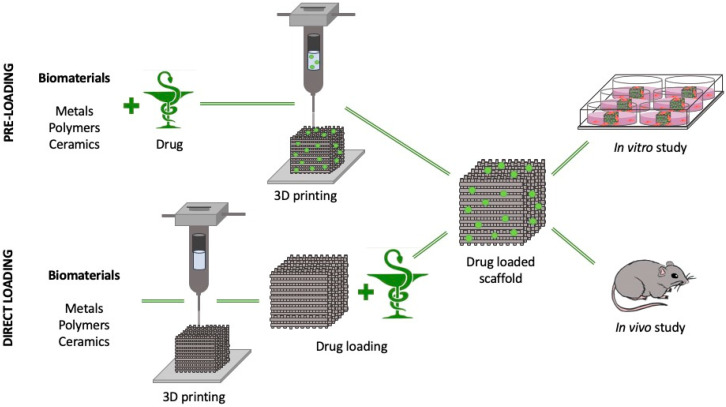
Schematic layout summarizing pre-loading and direct loading 3DP porous substrate fabrication for in vitro and in vivo drug delivery applications.

3DP technology can successfully assist engineers, pharmacologists and clinicians in the design and realization of 3D mesoporous scaffolds to be used for different medical applications such as tissue engineering and regenerative medicine implants characterized by the adjustable loading and unloading activity of pharmacologically active substances such as, antibiotics, growth and differentiation factors *(*Figure 2) [44].

These active substances can be incorporated inside the mesoporous 3D structures in two different main steps: during the manufacturing process (pre-loading, PL) by mixing the substances with the printable material and then proceeding with the 3DP technique in mild conditions (i.e., electrospinning or inkjet printing), or at the end of the printing step (direct loading, DL), by soaking the 3D-printed scaffold in a solution of the molecule to be loaded as reported for bone morphogenetic protein-2 (BMP-2) mesoporous calcium silicate (MesoCS) 3D-printed scaffold [45]. PL methods are usually applied for the production of scaffolds able to locally deliver antibiotiotics [46], but unfortunately, antibiotics such as those of the cephalosporin family have significantly reduced efficiency when exposed to heat and, consequently, the DL method is definitely applied to sensitive molecules when the 3DP process is carried out at high temperatures or pressures [47].

**Figure 3:**
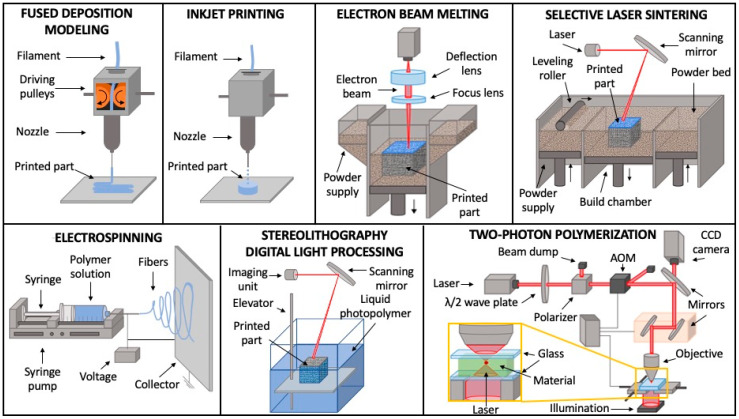
Schematic illustrations of the most diffused 3D printing fabrication techniques for porous scaffolds manufacturing: fused deposition modeling, inkjet printing, electron beam melting, selective laser sintering, stereolithography, electrospinning, two-photon polymerization.

There are many 3DP strategies available to the scientific community that allow the realization of mesoporous scaffolds under computer aids combining different processes and materials like carbon nanotubes, nanoparticles, nanofibers, polymers with active biomolecules with or without live cells. These 3DP fabrication techniques, as summarized in Figure 3, include fused deposition modeling (FDM), material jetting as inkjet printing (IP), electron beam melting (EBM), selective laser sintering (SLS), stereolithography (SLA) and digital light processing, electrospinning, and two-photon polymerization (TPP).

### 2.1. Fused Deposition Modeling

FDM is one of the most inexpensive nozzle-based deposition systems that allows direct printing of 3D CAD designed layer by layer objects. Thermoplastic degradable (polylactic acid, PLA, poly(ε-caprolactone), PCL, polyvinyl alcohol, PVA) and non-degradable (acrylo-nitrile butadiene styrene, ABS, ethylene vinyl acetate, EVA, poly methyl methacrylate, PMMA) polymer filament are pushed into the heater block to melt before extruding from a high-temperature nozzle solidifying onto the previous layer on the build plate [48].

The easiest method of loading target drugs into the thermoplastic polymer filament is the impregnation obtained leaving the just printed device in a concentrated drug solution (mostly ethanol or methanol) followed by a drying step [49,50].

### 2.2. Inkjet Printing

The inkjet-based non-contact printing technology reproduces digital patterns with tiny ink drops through thermal, piezoelectric and magnetic approaches. The thermal stimulation, reaching until 100–300 °C, nucleates a bubble and directly leads to droplet expulsion from the printhead. The size of droplets is related to the temperature gradient and ink viscosity employed. Likewise, the ink drop generation can be produced by the pulse strain and acoustic waves generated from a piezoelectric actuator and larger size ink droplets can be produced by means of electromagnetic filed [51,52,53].

### 2.3. Electron Beam Melting

EBM is a modern fast solution to manufacture metal parts on a layer-by-layer basis through an electron beam that, bombarding the metal powders, melts them, constructing 3D geometries. This technique compared with ones using a laser, are characterized by high energy utilization and material absorption rate, improved stability, and reduced maintenance fees [54]. Although this technique is successfully used for the realization of porous orthopedic and dental implants made of metallic biomedical alloys as Ti6Al4V [55], to date, there are no applications of EBM for the production of 3D printed mesoporous devices for drug delivery application. This is due to the fact that these kinds of scaffolds are characterized by large surface roughness since EBM microfabrication accuracy ranges from 0.3–0.4 mm.

### 2.4. Selective Laser Sintering

SLS operates without a mold through a computer-controlled laser beam, powder bed, a piston assuring a vertical movement, and a roller to spread continuously powder layers [55]. This technique allows the realization of polymeric, metallic, and ceramic parts. SLS implies solid and semisolid consolidation procedures at a sintering temperature usually lower than the melting point. In the semisolid process suitable for treating low melting point polymer, as PCL, polyglycolide, PLA and poly(l-lactic) acid (PLLA), partially melted powder particles produce a certain volume of the liquid phase, which glues other solid elements. Microsphere-based hydroxyapatite (HA)/PCL scaffolds realized by SLS, shows a highly ordered porous structure [56]. Polyamide/HA composite platforms with porosities ranging from 40% to 70% and with a maximum tensile strength of 21.4 MPa were obtained by SLS [55,57]. Although the low near-infrared laser absorptivity of oxide ceramics, the direct SLS of ceramics throughout powder coating adds to the low melting point or composites ceramics has been done [58]. Many sacrificial binders as waxes, thermoplastics, long-chain fatty acids or sometimes a combination of binders as thermoset/semi-crystalline PA-11 or wax/PMMA are used for the realization of porous 3D structured materials as graphite and composite ceramic Al_2_O_3_-ZrO_2_-TiC [59,60,61]. A high-energy laser beam increasing the temperature on the surface promotes the particle interaction to each other before sintering together, while the material on the grain borderline continues to diffuse into the pores, stimulating densification activities. Since SLS is characterized by a high heating rate and short holding time, it results as an excellent alternative in producing scaffolds supported by low-dimensional nanomaterials such as graphene and carbon nanotubes [62].

### 2.5. Electrospinning

Nanodimensional high specific surface area devices can be fabricated with bioactive loaded polymer through the electrospinning technique [63]. In a conventional electrospinning system, generally comprising of a high-voltage power supply, syringe, pump and a collector, polymeric nanofibers, inorganic nanofibers, and composite nanofibers are ejected into a sequence of droplets forming steady fiber [64,65].

### 2.6. Stereolithography and Digital Light Processing

Stereolithographic (SLA) and Digital Light Processing 3DP allow the layer by layer realization of 3D mesoporous stuff by cross-linking photo-sensitive materials using laser light or digital light projection technique, respectively [66]. The curing stereolithographic step, both in single-photon and two-photon polymerization, is actuated by tuning the incidence, the intensity, and the duration of near-infrared, visible or UV light.

### 2.7. Two-Photon Polymerization

While single-photon polymerization requires one-photon absorption, in TPP, the molecule simultaneously absorbs two photons. By employing a focused femtosecond near-IR, TPP stereolithography, processing biocompatible synthetic or natural hydrogels or polymers, grants the ultra-fast production of 3D structures with submicron resolution [67].

## 3. Applications of 3DP Mesoporous Material for Drug Delivery

Doing a search on the Web of Science and on Pubmed at the beginning of June 2020, resulted that in the last decades a fair number of publications are strictly related to the specific topic covered in this review and, more in details, related to mesoporous 3D printed materials for drug delivery applications. As highlighted in Table 1, most of the results focalized on the application of these 3D porous materials for tissue engineering bone substitute realization. Their porosity, by mimicking the bone structure, allows nutrient transport, waste removal, cell migration, angiogenesis and differentiation phenomena, assisting bone regeneration in bone defects related to traumatic events or pathologies.

### 3.1. Bone Regeneration

#### 3.1.1. Growth Factors and Peptides

Several FDA approved growth factors as BMP-2 have been used in clinics for bone and cartilage regeneration. In a mesoporous bioactive glass (MBG) covered silicate 1393 bioactive glass scaffolds candidate for bone repairing application, BMP-2 release was higher than that of DNA and dexamethasone. MBG successfully physically absorbed and released the active molecule without upsetting its pharmacological activity [68]. Fish hydrogel-based mesoporous strontium-doped calcium silicate scaffolds were proved to be efficient BMP-2 carriers for in vitro human Wharton jelly mesenchymal stem cell differentiation [69]. Customized 3D-printed osteoinductive implants were realized integrating porous silicon BMP-2 carriers within a 3D-printed PCL patient-specific implant [70]. FDM 3D MesoCS scaffolds combined with PCL were presented as odontoinductive biomaterial with efficient BMP-2 delivery capability [71]. In vitro tested BMP-2 pre-loaded mesoporous calcium silicate/PCL scaffolds, even if not suitable for clinical applications, exhibited high biocompatibility and sustained drug delivery pattern compared to the ones directly immersed with BMP-2 after the FDM fabrication [45].

As some authors reported some relevant side-effect related to the use of BMP-2 [72], 3D dipyridamole-coated hydroxyapatite (HA)/beta-tri-calcium phosphate (β-TCP) scaffolds were successfully used to promote bone regeneration in critical bone defects as well as BMP-2 [73].

Since vascularization is a key step of the osteogenesis process, 3DP dimethyloxallyl glycine loaded MBGs and poly(3-hydroxybutyrate-*co*-3-hydroxyhexanoate) polymers scaffolds and results showed that dimethyloxallyl glycine was effectively released improving angiogenesis and osteregeneration in the bone faults [74]. Vascular endothelial growth factor (VEGF), was well encapsulated in chitosan/dextran sulfate microparticles and mixed into a calcium phosphate paste for the 3D plotting of growth factor loaded calcium-phosphate-based scaffolds applicable for bone tissue engineering [75].

Materials for tissue regeneration can be functionalized with engineered peptides able to regulate bone healing and regeneration. In vitro tests with naringin and calcitonin gene-related peptide-loaded 3DP MBG/sodium alginate/gelatin scaffolds showed that their high porosity assure efficient sustained drug delivery [76]. Peptide osteostatin and Zn^2+^ ions loaded meso-macroporous 3D scaffolds based on MBGs, exhibited a synergistic effect improving human mesenchymal stem cell growth, promoting their osteogenic differentiation [77]. SLS 3DP poly(3-hydroxybutyrate) scaffolds, when post-printing loaded with osteogenic growth peptide, exhibited the ability to support cell growth and tissue restoration [78].

#### 3.1.2. Anti-Inflammatory and Antibiotics

Since any bone loss such as that following trauma, bone diseases and surgery, potentially provides suitable conditions for the onset of chronic infections or biofilm, it is highly desired the realization of anti-inflammatory and antibiotic-eluting scaffolds for sustained release without side effect in osteointegration, osteogenesis and osteoconduction processes. Dexamethasone loaded mesoporous CaSiO_3_/calcium sulfate hemihydrate (MCS/CSH) cement scaffolds have been realized by 3D printing. Compared to the tissue culture plates control, MCS/CSH scaffolds exhibited a good in vitro OCT-1 cells response, an extra balanced degradation rate and capacities to slowly release the uploaded drug in targeted sites [79].

3DP high porosity dual-drug delivery layered MBG/sodium-alginate (SA)–SA scaffolds were successfully fabricated enriching the printing step with SA cross-linking. They resulted able to stimulate proliferation and osteogenic differentiation of human bone marrow-derived mesenchymal stem cells, furthermore, bovine serum albumin (BSA) and ibuprofen were successfully loaded in SA layer and the MBG of MBG/SA layer, respectively, resulting in a quite fast BSA release due to the macroporous network of SA, and in a constant release of ibuprofen due to the retention effect of the mesoporous channels of MBG [80].

It is well-known that inflammation phenomena thwart bone regeneration in transplanted loci and the local effect of short-term corticosteroid administration increase the effectiveness of bone tissue engineering [81]. Dexamethasone-loaded polydopamine-functionalized MBG was incorporated into polyglycolic acid/poly-1-lactic acid (PGPL) to fabricate a 3D mesoporous scaffold via laser additive manufacturing able to stimulate cell differentiation, biomineralization [82]. Loaded dexamethasone electrospun fibrous scaffolds of PCL-gelatin, incorporating MBG nanoparticles (MBGn), were presented excellent valid 3D platforms for bone tissue engineering [83].

In the case of dexamethasone-loaded 3DP strontium-containing MBG scaffolds the mesoporous matrix with enhanced mechanical strength to ensure great bone-growing bioactivity together with marked drug delivery capability [84]. 3DP scaffolds realized by using MBG and concentrated alginate pastes efficiently delivered dexamethasone in an in vitro test with human bone marrow-derived mesenchymal stem cells thanks to their matrix characterized by a well-ordered network of nano-channels and micro and macro-pores [85]. Poly(1,8-octanediol-*co*-citrate) and β-tricalcium phosphate (β-Ca_3_(PO_4_)_2_), together with ibuprofen-loaded SiO_2_ were made-up by micro-droplet jetting 3DP technique. Their hierarchically macro/mesoporous extremely interconnected pore matrix made them a valid antimicrobial bioengineered solution for bone regeneration [86,87].

The antimicrobials local application usually provides higher drug delivery than those attained with the intravenous application [88,89] and many 3DP macro/meso-porous composite scaffolds, are at the moment used to support a reproducible safe a better and well-regulated in situ antibiotics delivery. Some doxorubicin-loaded 3DP magnetic Fe_3_O_4_ nanoparticles containing mesoporous bioactive glass/polycaprolactone composite scaffolds enhanced osteogenic activity also assured sustained local anticancer delivery coupled with magnetic hyperthermia treatment [90].

Multidrug-loaded scaffold undoubtedly improves the applicability of 3D rapid prototype implants to ward off biofilm growth and drug resistance. Antibiotics are usually locally delivered via PMMA bone cement spacers [91,92] compatible with a restricted number of antibiotics and characterized by having low release profiles. Mesoporous bioactive glass/metal-organic framework and macro/meso-porous composite bioactive ceramics bound with poly (3-hydroxybutyrate-*co*-3-hydroxyhexanoate) scaffolds loaded with high dosages of isoniazid and/or rifampin, anti- tuberculosis drugs, had good biocompatibility and bioactivity when tested for long-term therapy after osteoarticular tuberculosis debridement surgery. Hierarchical 3DP multidrug scaffolds built with nanocomposite bioceramic and PVA were coated of gelatin-glutaraldehyde (Gel-Glu). Levofloxacin was loaded into the mesopores of the bioceramic part, vancomycin was packed into the biopolymer portion while rifampin in the external layer of Gel-Glu. The early delivery of rifampin followed by a sustained release of vancomycin and levofloxacin, represented an excellent and encouraging alternative for bone infection management [93]. 3DP rifampin- and vancomycin-loaded calcium phosphate scaffolds, used in a mouse model implant-associated staphylococcus aureus bone infection, proved that the concomitant local delivery of rifampin and vancomycin significantly improves the outcomes of the implant compared to PMMA spacers which cannot carry rifampin [94]. Gelatine and Si-doped hydroxyapatite porous 3D scaffolds were successfully loaded with vancomycin since they were rapidly prototyped fabricated at room temperature and apart from by increasing in vitro pre-osteoblastic MC3T3-E1 cell differentiation they also inhibit bacterial growth [95].

#### 3.1.3. Metallic Ions and Trace Elements

Recently, many metallic ions such as zinc, copper, silver, cerium, strontium and cobalt, were combined with bioactive glasses to improve osteogenesis and angiogenesis [96,97,98]. Silver, among all, is the one that stands out for its strong antibacterial qualities. Silver/graphene oxide homogeneous nanocomposites were modified on 3DP β-tricalcium phosphate bioceramic scaffolds leading to a bifunctional scaffold with, just test in vitro, antibacterial and osteogenic activity were realized and in vitro tested [99].

In addition to the direct effect that a drug-loaded on a scaffold can have at the implantation site, several authors highlighted that also the integration of trace elements such as strontium, zinc, magnesium, calcium, copper, boron and cerium in 3DP mesoporous bioactive glass scaffolds enhance in vitro and in vivo osteogenic and differentiation activity [100,101,102].

### 3.2. Other Applications

Apart from the numerous applications in the bone regeneration field, mesoporous 3DP scaffolds including also mesoporous elastomer characterized by ordered and aligned nanofibrillar architecture that can be rapidly managed into multifaceted objects are starting to be more and more widespread even in other branches of biomedical research and medical clinic [103].

Coaxial electrospinned silk fibroin-based scaffolds are successfully tested as a potential brain-derived neurotrophic factor and VEGF delivery carrier in nerve repair and reconstruction applications [104].

Anti-HIV-1 drugs, including emtricitabine, tenofovir disoproxil fumarate and efavirenz were successfully loaded in a 24-layered rectangular prism-shaped 3DP controlled release fixed-dose combination tablets able to control the intestinal release of the active molecules [105].

## 4. Conclusions

For many years, 3D devices have been assisting research in very different areas, ranging from simple cell cultures to tissue engineering and drug delivery applications. 2D cell culture represents a chief tool in molecular and cellular biology due to its fast, ease, reproducibility and cheap distinctive characteristic. However, it is now universally accepted that 2D cell culture methods understate the live cells in vivo setting unlike reported for last-generation 3D biomaterials which, on the contrary, are able to mimic in a much more realistic way the environment required for a whole range of biomedical and clinical applications. The development of three-dimensional supports has even greater resonance in tissue engineering and regenerative medicine applications since, in those cases, the function of tissues or organs must be restored ensuring the spatial and functional interconnection between different cell types, in order to guarantee the exchange of gas, nutrients or drugs and the elimination of waste products. In this review, we wanted to highlight how these characteristics can be optimized by merging together the need to provide solid supports capable of assisting cell growth at the level of tissue and organ and, at the same time, the right degree of porosity of the materials that in the specific case of 3D biomaterials offers a whole series of drug delivery capabilities worthy of study and implementation. The way an active molecule is carried to a specific region or cellular type can impact on its interaction efficacy. Each drug has a therapeutic window in which health benefits must be maximized and side effects minimized. This need has materialized in the ever-stricter demand of a multidisciplinary approach for the implementation of new materials and methods for an effective in vitro and in vivo drug delivery. Materials science, chemistry and micro/nanofabrication offer both original and effective solutions applicable in research and clinical areas. The rapid and often inexpensive fabrication of 3DP structures enhances the performance of devices no longer used only as structural supports for tissue regeneration and differentiation thanks to the optimization of their intrinsic and tuneable porosity. 3DP mesoporous devices allow an effective drug delivery of personalized therapy, customizable both from the geometric point of view and from the point of view of pharmacological requests for each individual patient. With the topics covered in this review, we want to highlight how 3D printing techniques allow the production of CAD designing structures that fully correspond to the request of each patient in response to needs following trauma or pathologies. The future implementation of new biodegradable biopolymers and of multi-step etching processes for post-printing functionalization/modification, will also allow more efficient drug delivery application of scaffolds as the 3D EBM produced ones, by conferring them the not-yet optimized degree of mesoporosity.

## Figures and Tables

**Table 1:** Applications of 3DP porous materials for tissue engineering and bone substitute realization.

Material	3D Printing Method	Drug	Drug Loading Method	Application	Reference
Mesoporous strontium substitution calcium silicate/recycled fish gelatin 3D cell-laden scaffold.	IP	BMP-2	DL	Bone tissue engineering.	[69]
				In vitro	
PCL 3DP patient-specific implant, with degradable porous silicon-based carriers.	SLA		DL	Bone graft for critical size bone defects.	[70]
				In vitro	
Mesoporous calcium silicate 3DP scaffold.	FDM		DL	Bone regeneration.	[45]
				In vitro	
Hierarchical 3D multidrug scaffolds based on nanocomposite bioceramic and PVA with an external coating of gelatin-glutaraldehyde.	IP	Dipyridamole and BMP-2	DL	Bone tissue engineering.	[73]
				In vivo (mice)	
Scaffold is composed of MBG and poly(3-hydroxybutyrate-*co*-3-hydroxyhexanoate) polymers.	IP	Dimethyloxallyl glycine	PL	Angiogenesis and osteogenesis for bone tissue engineering.	[74]
				In vivo (rats)	
MBG with sodium alginate and gelatin.	IP	Naringin and calcitonin gene-related peptide	DL	Bone repair.	[76]
				In vitro	
MBG.	IP	Peptide osteostatin and Zn^2+^ ions	DL	Bone grafts with enhanced osteogenic capacity.	[77]
				In vitro	
Poly(3-hydroxybutyrate) scaffold.	SLS	Osteogenic growth peptide and its C-terminal sequence (10–14)	DL	Bone tissue engineering.	[78]
				In vitro	
Calcium phosphate cement scaffolds by 3D plotting with growth factors encapsulating chitosan/dextran sulfate microparticles mixed into the paste.	IP	BSA and VEGF	PL	Encapsulate growth factors in a cement.	[75]
				In vitro	
Layered MBG/SA.	IP	BSA and ibuprofen	PL	Stimulate human bone mesenchymal stem cells (hBMSCs) adhesion, proliferation and osteogenic differentiation.	[80]
				In vitro	
Integrate MBG with 3D printing basic 1393 bioactive glass scaffolds.	IP	Dexamethasone and BMP-2	DL	Bone repair and relative bone disease treatment.	[68]
				In vivo (rats)	
MBG is functionalized with polydopamine and PGPL.	SLS	Dexamethasone	PL	Osteogenic differentiation and biomineralization.	[82]
				In vitro	
Calcium sulfate hemihydrate cement is incorporated in mesoporous calcium silicate.	IP		PL	Bone tissue engineering.	[79]
				In vitro	
Electrospun fibrous scaffolds of PCL-gelatin incorporating mesoporous bioactive glass nanoparticles.	ES		PL	Bone regeneration.	[83]
				In vivo (rats)	
MBG with strontium.	IP		PL	Bone regeneration.	[84]
				In vitro	
Hierarchical scaffolds of MBG and concentrated alginate pastes.	IP		PL	Bone tissue engineering.	[85]
				In vitro	
3D magnetic Fe_3_O_4_ nanoparticles containing MBG/PCL composite scaffolds.	IP	Doxorubicin	PL	Osteogenic activity, local anticancer drug delivery and magnetic hyperthermia.	[90]
				In vitro	
Hollow mesoporous structure of silica (SiO_2_) microspheres loaded in a Poly(1,8-octanediol-*co*-citrate) and β-tricalcium phosphate scaffold.	IP	Ibuprofen	PL	Bone regeneration of infected bone defects.	[86]
				In vitro	
Poly(1,8-octanediol-*co*-citrate) and β-tricalcium phosphate scaffold.	IP		PL	Bone defect repair.	[87]
				In vitro	
MBG with MOFs and PCL.	IP	Isoniazid	PL	Osteoarticular tuberculosis treatment.	[106]
				In vitro	
Carboxylic MBG and methyl-functionalized mesoporous silica nanoparticles.	IP	Isoniazid and rifampin	PL	Filler after surgical treatment of osteoarticular tuberculosis.	[107]
				In vivo (rabbits)	
Hierarchical 3D multidrug scaffolds based on nanocomposite bioceramic and PVA with an external coating of Gel-Glu.	IP	Rifampin, levofloxacin and vancomycin	PL	Destroy Gram-positive and Gram-negative bacteria biofilms for local bone infection therapy.	[93]
				In vitro	
3D printed calcium phosphate scaffolds.	IP	Rifampin and vancomycin	PL	Treat an implant-associated Staphylococcus aureus bone infection.	[94]
				In vivo (mice)	
Porous 3-D scaffolds consisting of gelatine and Si-doped hydroxyapatite.	IP	Vancomycin	PL	Pre-osteoblastic MC3T3-E1 cell differentiation.	[95]
				In vitro	
β-tricalcium phosphate bioceramic scaffolds with a homogeneous nanocomposite made of silver nanoparticles and graphene oxide.	IP	Silver nanoparticles and graphene oxide	PL	Bone grafts with good antibacterial performance.	[99]
				In vitro	
Borosilicate MBG.	IP	Boron and silicon ions	PL	Repair bone defects.	[100]
				In vivo (rats)	
3D porous composite scaffolds made of cerium oxide, mesoporous calcium silicate and PCL.	IP	Cerium ions	PL	Bone regeneration.	[101]
				In vitro	
MBG with strontium.	IP	Strontium ions	PL	Bone regeneration.	[102]
				In vivo (rats)	
MBG modified β-tricalcium phosphate.	IP	Calcium, phosphorus and silicon ions	PL	Angiogenesis and osteogenesis for bone tissue engineering.	[108]
				In vivo (rabbits)	
Mesoporous calcium silicate 3D-printed scaffold.	IP	BMP-2	PL	Odontoinductive biomaterial in regenerative endodontics.	[71]
				In vitro	
Silk fibroin porous scaffold.	ES	Brain-derived neurotrophic factor and VEGF	PL	Cavernous nerve regeneration.	[104]
				In vivo (rats)	
Nanostructured ordered mesoporous elastomers composed of molecular double networks (poly(ethylene oxide)–poly(propylene oxide)–poly(ethylene oxide) pluronic copolymers, and PMMA.	IP	Ibuprofen and vancomycin	DL	Biomedical and engineering applications as the need for high mechanical performance coexisting with precise nano-microstructural features.	[103]
				In vitro	
Humic acid-polyquaternium 10 tablet.	IP	Efavirenz, tenofovir disoproxil fumarate and emtricitabine	PL	Anti-HIV-1 controlled drug delivery.	[105]
				In vivo (pigs)	
Mesoporous iron oxide nanoraspberry inside microneedles.	DLP	Minoxdil	DL	Treatment of androgenetic alopecia.	[109]
				In vivo (mice)	
Porous poly(ethylene glycol) dimethacrylate devices.	TPP	Rhodamine B as model drug	PL	Different biomedical applications.	[110]
				In vitro	
